# Study on adolescents’ attitudes and attachment toward companion animals: mitigating the negative effects of cultural estrangement on wellbeing

**DOI:** 10.3389/fpsyg.2025.1552127

**Published:** 2025-04-11

**Authors:** Hikari Koyasu, Sakura Ogasawara, Takefumi Kikusui, Toshiya Murai, Atsushi Nishida, Miho Nagasawa

**Affiliations:** ^1^Laboratory of Human-Animal Interaction and Reciprocity, Department of Animal Science and Biotechnology, Azabu University, Sagamihara, Kanagawa, Japan; ^2^Department of Psychiatry, Graduate School of Medicine, Kyoto University, Kyoto, Japan; ^3^The Unit for Mental Health Promotion, Research Center for Social Science & Medicine, Tokyo Metropolitan Institute of Medical Science, Tokyo, Japan

**Keywords:** mental health, cultural estrangement, adolescent, companion animals, attachment

## Abstract

The mental health of adolescents is an important issue, since it has a significant impact on their future lives. In this regard, one of the methods for supporting adolescents’ mental health is the interaction with companion animals, which is becoming widely recognized. In our previous research, we showed that owning companion animals has positive effects on adolescents’ wellbeing and cultural estrangement. However, the effect was notably small. The findings of studies examining companion animals and mental health are inconsistent. These results suggest the need to not only focus on pet ownership, but also how their relationship with companion animals such as attitudes toward animals and attachment. In addition, the impact of cultural estrangement on wellbeing is not always negative, and their relationship may not be a simple correlation. Therefore, this study focused on individuals with high cultural estrangement and aimed to clarify how attitudes toward animals and attachment to their pets differ depending on whether their wellbeing is high or low. Based on the results, the group with high cultural estrangement and high wellbeing exhibited an anthropocentric attitude and a strong interest in nature and ecology. In addition, the group with high cultural estrangement and high wellbeing exhibited close attachment to their pets, which functioned as confidants. This finding highlights the specific role of such animals in supporting adolescents’ mental health during this critical developmental stage.

## 1 Introduction

Adolescence is an important stage in life characterized by rapid psychological, social and neurobiological development (Steinberg and Morris, 2001). During this period, the formation of values and social skills are fostered (Waterman, 1982; Adams and Marshall, 1996; Kilford et al., 2016), which have a significant impact on future interpersonal relationships and mental health (Johnson et al., 2009; Nishida et al., 2016; Schlack et al., 2021; Copeland et al., 2009; Kim-Cohen et al., 2003; Fergusson et al., 2005; Copeland et al., 2013). In addition to these developments, adolescents experience anxiety related to their social environment (Greca and Lopez, 1998). In fact, 10–20% of children and adolescents worldwide experience mental health issues, with numbers that are steadily increasing (Kieling et al., 2011). Thus, it is strongly recommended that preventive interventions and multi-faceted support for adolescents be promoted to maintain their mental health (Dahl et al., 2018).

Previous research has explored two main approaches to the relationship between values and wellbeing: the direct effects of value priorities and the fitness between personal and environmental values. The first approach suggests that pursuing intrinsic values (e.g., self-acceptance, affiliation) enhances wellbeing, while extrinsic values (e.g., material success, fame) harm it, with only weak direct effects on the affective aspects of wellbeing (Sagiv and Schwartz, 2000; Kasser and Ryan, 1996; Vansteenkiste et al., 2006). The second approach emphasizes the congruity between personal values and environmental values, arguing that such alignment promotes wellbeing beyond the modest direct effects of values (Sagiv and Schwartz, 2000). However, conflicting findings challenge the importance of congruity, with studies showing that extrinsically oriented individuals may experience lower wellbeing regardless of value-environment alignment (Kasser and Ahuvia, 2002; Vansteenkiste et al., 2006). Furthermore, another research on cultural estrangement did not replicate the importance of value congruence either (Bernard et al., 2006). Cultural estrangement has typically been operationalized as an individual’s rejection of, or sense of removal from, dominant social values and beliefs (Kohn et al., 1984; Seeman, 1991). Bernard et al. (2006) findings showed that there was no significant relation between value discrepancies and low psychological outcomes. These relationships between value congruence and wellbeing may not be as straightforward as originally proposed.

It is becoming widely recognized that one aspect of mental health support is involvement with companion animals. While there have been reported the positive effects of companion animals on children’s and adolescents’ mental health (Black, 2012; Gadomski et al., 2015; Lem et al., 2016), some studies have indicated negative effects or no significant association (Miles et al., 2017; Żebrowska et al., 2023; Gilbey et al., 2007; Koivusilta and Ojanlatva, 2006; Gillum and Obisesan, 2010), or mixed effects dependent on pet species (Endo et al., 2020). For an example of positive effect, adolescents who owned pets felt less loneliness than those who did not (Black, 2012). For an example of negative effect, pet owners were found to be physically healthier than non-pet owners, they also had psychological problems such as anxiety, chronic fatigue, insomnia, and depression (Müllersdorf et al., 2010). The impacts of companion animals on adolescents’ mental health are inconsistent. In our previous research (Koyasu et al., 2023), we have also found that owning a dog or cat had a positive effect on adolescents’ wellbeing and cultural estrangement through relationships with others such as family members. However, the effect was notably small.

In this regard, there are two critical issues from previous research that must be addressed. First, there is the possibility that the effect of companion animals on adolescents’ mental health depends on the role that they play in adolescents’ lives. The relationship with animals is diverse; while many animals are valued for economic and practical reasons, modern companion animals are valued for the benefits from their relationship with humans. It is important to focus on the relationship between companion animals and adolescents. There is a wide range of attitudes toward animals, for instance, *naturalistic* - primary interest in and affection for wildlife, *humanistic* - primary interest in and strong affection for individual animals, principally pets, *moralistic* - primary concern for the right and wrong treatment of animals, with strong opposition to exploitation of and cruelty toward animals, *utilitarian* - primary concern for the practical and material value of animals, and *negativistic* - primary orientation an active avoidance of animals due to dislike or fear (e.g., Kellert, 1982; Kellert, 1991; Ishida et al., 1991; Kellert, 2002; Su, 2018) People with a strong *humanistic* attitude tend to have an emotional attachment to their pets and interact with them anthropomorphically. Conversely, people with a *dominant* attitude tend to be more interested in controlling and exerting power over animals. These contrasting attitudes affect how people interact with and form attachments to their pets. For example, when a *familial* attitude is prominent, it is expected that a deeper bond and attachment to companion animals will be established, while the emotional support provided by the animals will also be enhanced. Additionally, viewing pets as family members has been shown to improve wellbeing (Mcconnell et al., 2019). In contrast, for people with a high *utilitarian* or *indifferent* attitude, it may have limited effects of companion animals on mental health, even if they own.

It has also been indicated that the degree of attachment to their pets has an effect on mental health, loneliness, and self-esteem (Paul and Serpell, 1996; Black, 2012; Hartwig and Signal, 2020; Marsa-Sambola et al., 2017; Triebenbacher, 1998; Hawkins et al., 2024). Rather than solely focusing on ownership, attention should be given to the relationship between adolescents and pets. Moreover, attitudes toward animals significantly vary across cultures (Sinclair et al., 2022). For instance, Japanese people tend to have strong psychological and emotional attachment to their pets (Kellert, 1991; Ishida et al., 1991). This specific attitude toward animals is believed to contribute to mental health and wellbeing. This provides essential insights for considering culturally rooted approaches to enhancing mental health.

The second issue is the linear approach in previous research that assessed the relationship between cultural estrangement and mental health. Since the perceptions of cultural estrangement can vary (depending on the individual), it is not always a negative experience (Benish-Weisman et al., 2020, Flurry et al., 2021, Sortheix and Lönnqvist, 2015). Even when individuals’ values are not aligned with their environment, mental health could be maintained at a high level, if they have high self-esteem or have others with whom they can share their concerns. In fact, it has been shown that the presence and number of confidantes reduces depressive symptoms, also in adolescents (Nishida et al., 2024; Hall-Lande et al., 2007). As companion animals may serve to supplement the benefits usually derived from interpersonal relationships (Veevers, 1985), they could also function as a confidant.

Therefore, we propose a hypothesis in which individuals who maintain high mental health despite having strong cultural estrangement tend to get psychological support through close relationships with pets. This study focused on individuals with high cultural estrangement and aimed to clarify how their relationship with companion animals differs depending on whether their wellbeing is high or low.

## 2 Methods

**Figure 1:**
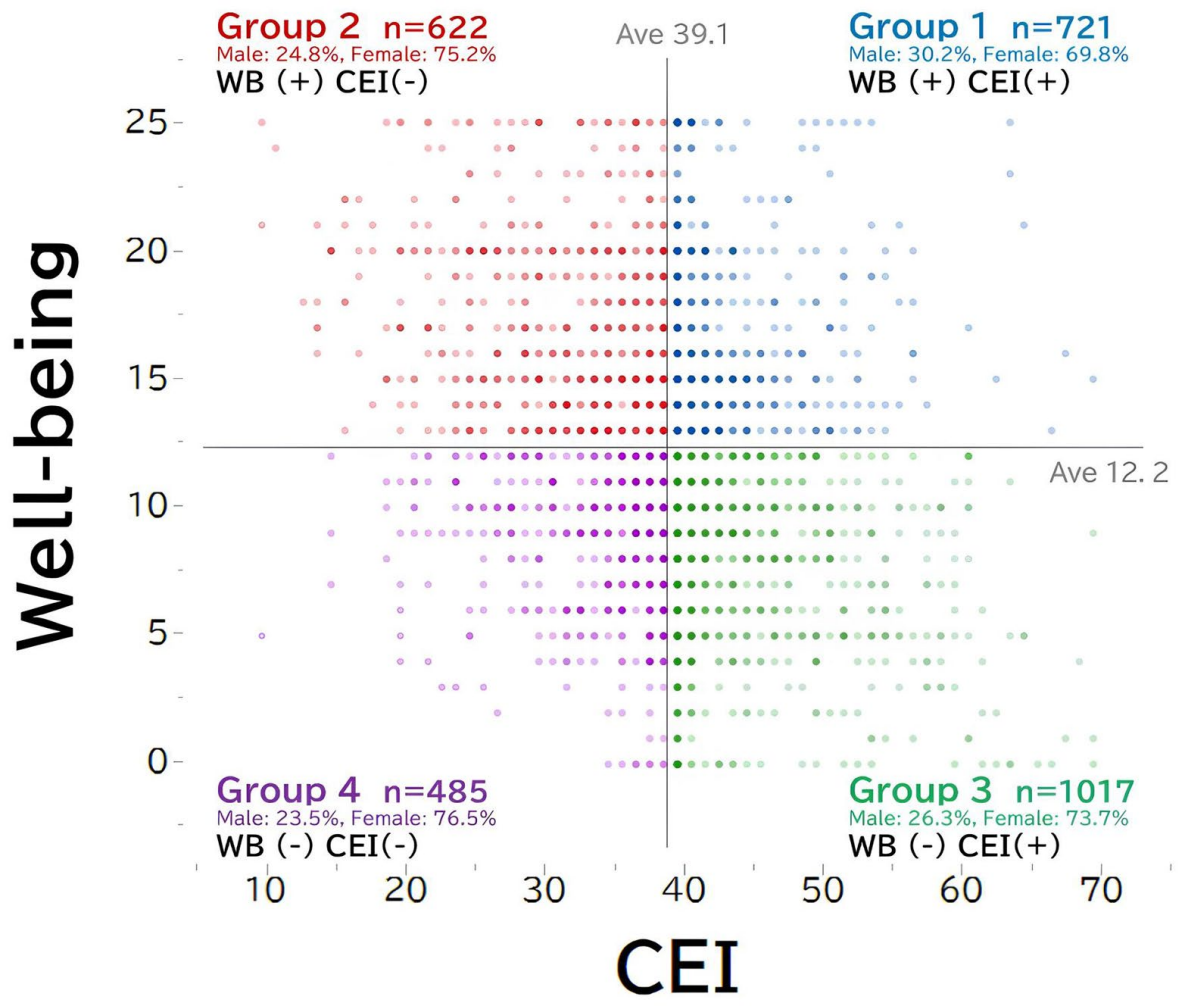
The four groups of cultural estrangement inventory (CEI) and wellbeing. The groups were divided according to the mean values for cultural estrangement and wellbeing.

In this study, the four-quadrants matrix of cultural estrangement and wellbeing were created. In this way, participants were divided into four groups: high cultural estrangement and high wellbeing, high cultural estrangement and low wellbeing, low cultural estrangement and high wellbeing, and low cultural estrangement and low wellbeing (Figure 1). In addition, the attitudes toward animals, the status of ownership, and the degree of attachment were compared between the group with high cultural estrangement and high wellbeing and the group with high cultural estrangement and low wellbeing.

### 2.1 Questionnaires and participants

To collect a large number of responses and uncover general trends, data from a questionnaire survey was used. The questionnaires were administered online, and the data was collected through Cross Marketing Inc., which is survey company (Tokyo, Japan). The data was then screened through the survey company to target a sample of high school and university students. During the transition from late adolescence to adulthood, people are more likely to have interpersonal problems as they move beyond family relationships to a society in which relationships with friends or others, and they become involved with a diverse variety of people (Zarrett and Eccles, 2006). After screening, the sample consisted of 1,033 high school students and 1,812 university students, for a total of 2,845 students (753 males and 2,092 females).

### 2.2 Contents

We re-analyzed the data from Koyasu et al. (2023). The questionnaires included basic demographic information, pet ownership status, attitudes and attachment toward companion animals, and views of such animals as indicators of pet-relatedness. The data on attitudes toward animals and attachment to pets were not used in previous research and were used for the first time in the analysis in this study. In this study, the Cultural Estrangement Inventory (CEI) and the World Health Organization-Five (WHO-5) Well-Being Index were used. The CEI was employed to assess the congruence between an individual’s values and the values emphasized in his/her environment. The WHO-5 scale was adopted as a measure of mental health because it is a very simple scale with five items, and it has both reliability and validity.

### 2.3 Basic demographic information

All the participants provided their gender, age, prefecture of residence, education (high school/university), type of residence (condominium/apartment complex, etc.), household income, and family composition.

### 2.4 Relationships with animals

We used their views on animals, their ownership status, and their attachment to pets. We used these measures to explore the adolescent’s relationship with animals and what animals, especially companion animals, mean to them.

#### 2.4.1 Attitudes toward animals

Attitudes toward animals were modified into a Japanese version by Ishida et al. (1991), which was based on Kellert’s (1974) study. The questionnaire consisted of 46 items based on a five-point Likert scale.

#### 2.4.2 Ownership of companion animals

The participants provided information on their current and previous pet ownership experiences, the type of pet (dog, cat, or other), where they kept their dog/cat, and the amount of time they spent interacting with the dog/cat before and during elementary school, middle school, high school, and college. It should be noted that since dogs and cats were the most common companion animals, only dog and cat owners were defined as companion animal owners.

#### 2.4.3 Attachment to pets

The level of attachment to pets was measured by using Kaneko’s (2018) scale, which focused on the multi-dimensions of attachment. It consists of 10 items regarding basic attachment and dependent attachment (referred to as “close attachment” in the present study), scored on a five-point Likert scale. The representative items for basic attachment were “I feel at ease when I am with my pet” and “My pet makes me feel happy.” The representative items for close attachment were “I often share important matters or confide my feelings with my pet” and “I feel closer to my pet than to any of my family members.” In this case, Cronbach’s alpha coefficient was 0.838 for basic attachment and 0.726 for close attachment.

### 2.5 Cultural estrangement inventory (CEI)

The CEI, developed by Cozzarelli and Karafa (1998), was used to examine the extent to which the participants felt that their own values were consistent with those of their family members and surrounding environment. Specifically, this scale measures the level of cultural estrangement and consists of 10 items regarding cultural atypical and misfit. In the present study, to examine the degree to which individual values are matched with the surrounding environment, the item “The Japanese and people in this country” was replaced with “Friends and local people.” It was adapted to examine cultural estrangement in societies that are more familiar to high school and university students. The responses were based on a seven-point Likert scale. Following previous research (Cozzarelli and Karafa, 1998), atypical and misfit subscales and total values were calculated. Atypical consisted of four items (representative items: “I feel that my opinions in important matters are similar to the opinions of typical or average my friends and local people” and “I strongly identify with value of my friends and local people,” all items were reverse), while misfit consisted of five items (representative items: “I often feel that somehow I do not fit in” and “I feel as though most my friends and local people do not understand me.”). Cronbach’s alpha coefficient for all 10 items was 0.821, for atypical was 0.877 and for misfit was 0.883. The Total value was used in this paper.

### 2.6 World Health Organization-five wellbeing index (WHO-5)

The World Health Organization-Five Well-Being Index (WHO-5) was used as a simple indicator of mental health over the past 2 weeks. The advantage of this index is its ability to measure mental health in a short time. The Japanese version of the WHO-5 was translated by Awata et al. (2007), after confirming equivalence with the original version and undergoing standardization procedures. The WHO-5 consists of five items that focus on the participants’ moods in daily life (e.g., “During the last 2 weeks, have you been in a cheerful and pleasant mood?”), with responses based on a five-point Likert scale. The overall score was calculated from the total of the five items, with a score of zero indicating the lowest wellbeing and a score of 25 indicating the highest wellbeing. In this case, Cronbach’s alpha coefficient was 0.910.

### 2.7 Statistical analysis

To determine whether there was a difference in the pet ownership rate in the groups, a cross-tabulation table was created for the number of people in each group who owned a dog or cat. In this case, a chi-square test was used to test whether there was a difference in the rate of dog or cat ownership. For the items in which the *chi-square test* showed significant differences, a *chi-square test* for each pair was conducted (corrected by using the *Bonferroni method*). In addition, to examine the effect of the relationship with pets, an analysis of variance (*ANOVA*) was conducted with attitudes toward animals and attachment as the objective variables and the group as the explanatory variable. If there was a significant effect, then a *t-test* was used to perform multiple comparisons. For such comparisons, the *Bonferroni method* was used to correct the significance level.

For the attitudes toward animals, *exploratory factor analysis* was conducted by using the *maximum likelihood method* (Var*imax rotation*). The number of factors was determined to be three based on the screen plot and the *Kaiser-Guttman criterion*, and the factor scores were estimated using the Thurstone method. The model fit indices were *RMSEA* = 0.049 and *TLI* = 0.879. The factors were named independently from those used by Ishida *et al*.: close relationships, anthropocentric, and interest in the environment and ecology (see Supplementary Information). The representative items for close relationships factor were “If I will have a pet, I would truly want to have it as a member of the family,” “I think having a pet is good for our health” and “Having a pet enriches human life.” The representative items for anthropocentric factor were “I think it is acceptable to destroy wild dogs,” “Taking euthanasia for granted, if animals become too numerous” and “I have no objection to creating pets that suit human preferences through selective breeding.” The representative items for interest in the environment and ecology factor were “I am interested in the morphology and classification of animals,” “I want to go to the mountains to see wildlife” and “I am interested in ecology and ecology-related topics.” These three factors were used in subsequent group comparison analyses. Meanwhile, analyses of the effects of attachment were limited to the data from companion animal owners (including current and previous owners).

## 3 Results

The descriptive statistics of the participants, such as gender and percentage of dog or cat ownership, are presented in Table 1.

**Table 1:** Descriptive statistics.

	Mean	SD
Age	18.70	2.22
WHO-5 wellbeing	12.21	5.81
Cultural estrangement		
Total	39.42	7.68
Atypical	20.44	5.56
Misfit	15.18	5.56

	Proportion (%)
Gender	
Male	26.47
Female	73.53
Ownership	
Dogs_current	13.92
Cats_current	8.08
Dogs_previous	14.59
Cats_previous	8.61
	*N* = 2,781

### 3.1 Attitudes toward animals

**Figure 2:**
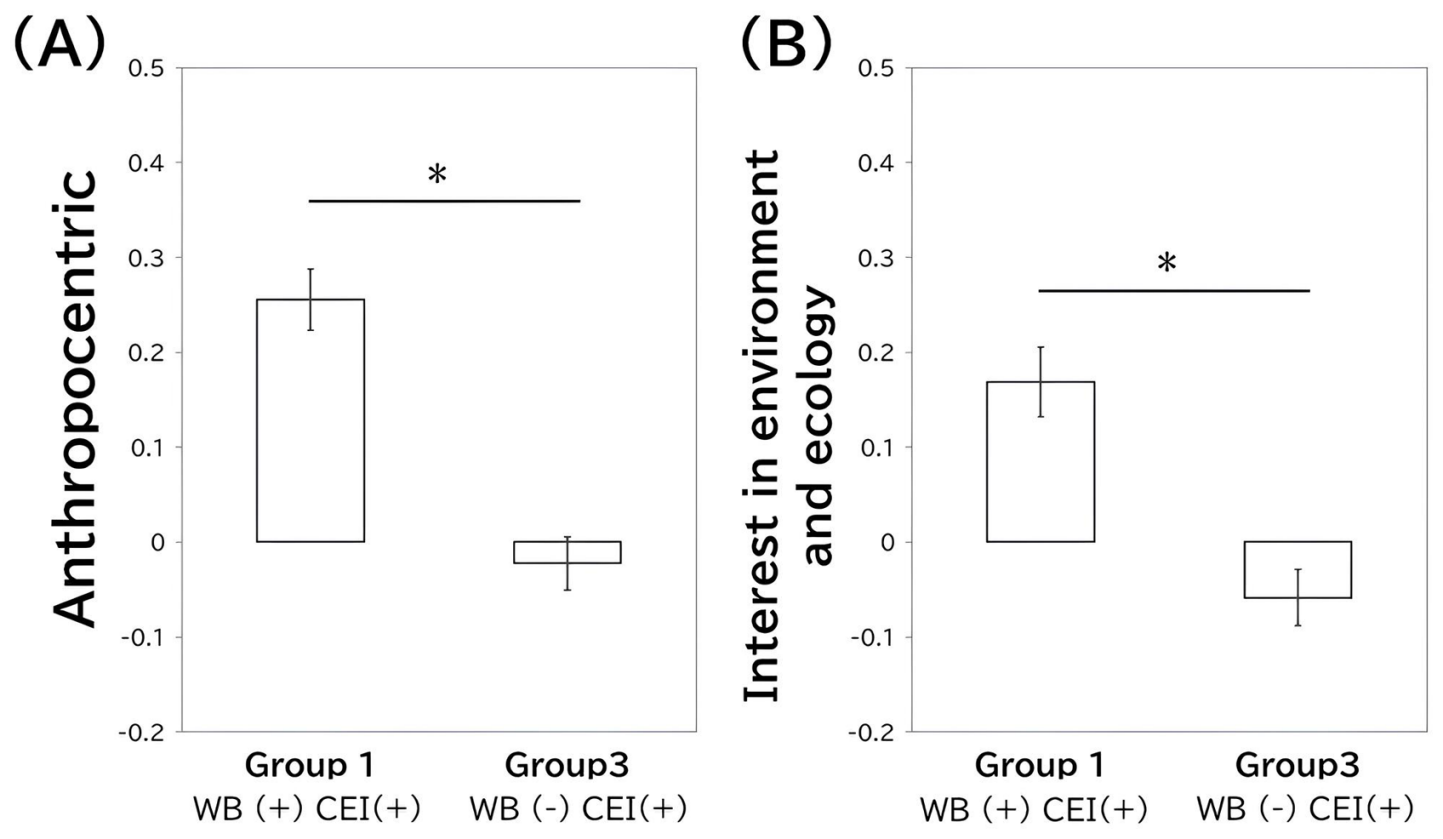
Differences between Groups 1 and 3 in the anthropocentric and interest in the environment and ecology categories. Group 1 comprised those with high cultural estrangement and high wellbeing, while Group 3 comprised those with high cultural estrangement and low wellbeing. Asterisks indicate *p* < 0.05. **(A)** Shows the anthropocentric values, while **(B)** shows the interest in environment and ecology values. The bars indicate the mean, and the error bars indicate the standard errors. CEI indicates cultural estrangement and WB indicates wellbeing.

In this study, we compared the three factor scores for attitudes toward animals—close relationships, anthropocentric views, and interest in the environment and ecology—across four groups defined by the CEI and wellbeing categories (see Table 2). *ANOVA* revealed significant differences for all factors: close relationships [*F*(3, 2,841) = 24.508, *p* < 0.001], anthropocentric [*F*(3, 2,841) = 28.891, *p* < 0.001], and interest in the environment and ecology [*F*(3, 2,841) = 10.655, *p* < 0.001]. Subsequent multiple comparisons showed that, for close relationships, Group 2 scored higher than Group 3, Group 1, and Group 4 (all *p* < 0.001). In anthropocentric views, Group 1 had higher scores than Groups 2, 3, and 4 (all *p* < 0.001), while Group 3 scored higher than Group 4 (*p* < 0.001). Regarding interest in the environment and ecology, Group 1 scored higher than all other groups (all *p* < 0.001). To summarize the differences between Group 1 and Group 3, which are the focus of this study, Group 1 had significantly higher scores in both anthropocentric and interest in the environment and ecology (Figure 2).

**Table 2:** Comparison of the attitudes toward animals between the four groups of CEI and wellbeing.

Factor of attitude toward animals	Chi-squared test		Multiple comparisons					
	Chi-square	*p*	1 vs. 3	2 vs. 4	1 vs. 2	1 vs. 4	2 vs. 3	3 vs. 4
Close relationships	24.508	<0.001		2 > 4 **	2 > 1 **		2 > 3 **	4 > 3 **
Anthropocentric	28.891	<0.001	1 > 3 **		1 > 2 **	1 > 4 **		3 > 4 **
Interest in the environment and ecology	10.655	<0.001	1 > 3 **		1 > 2 **	1 > 4 **		

Multiple comparison results show comparisons between groups (** *p* < 0.001). Bold text indicates comparisons between Groups 1 and 3, which were the focus of this study in particular.

### 3.2 Ownership of companion animals

**Figure 3:**
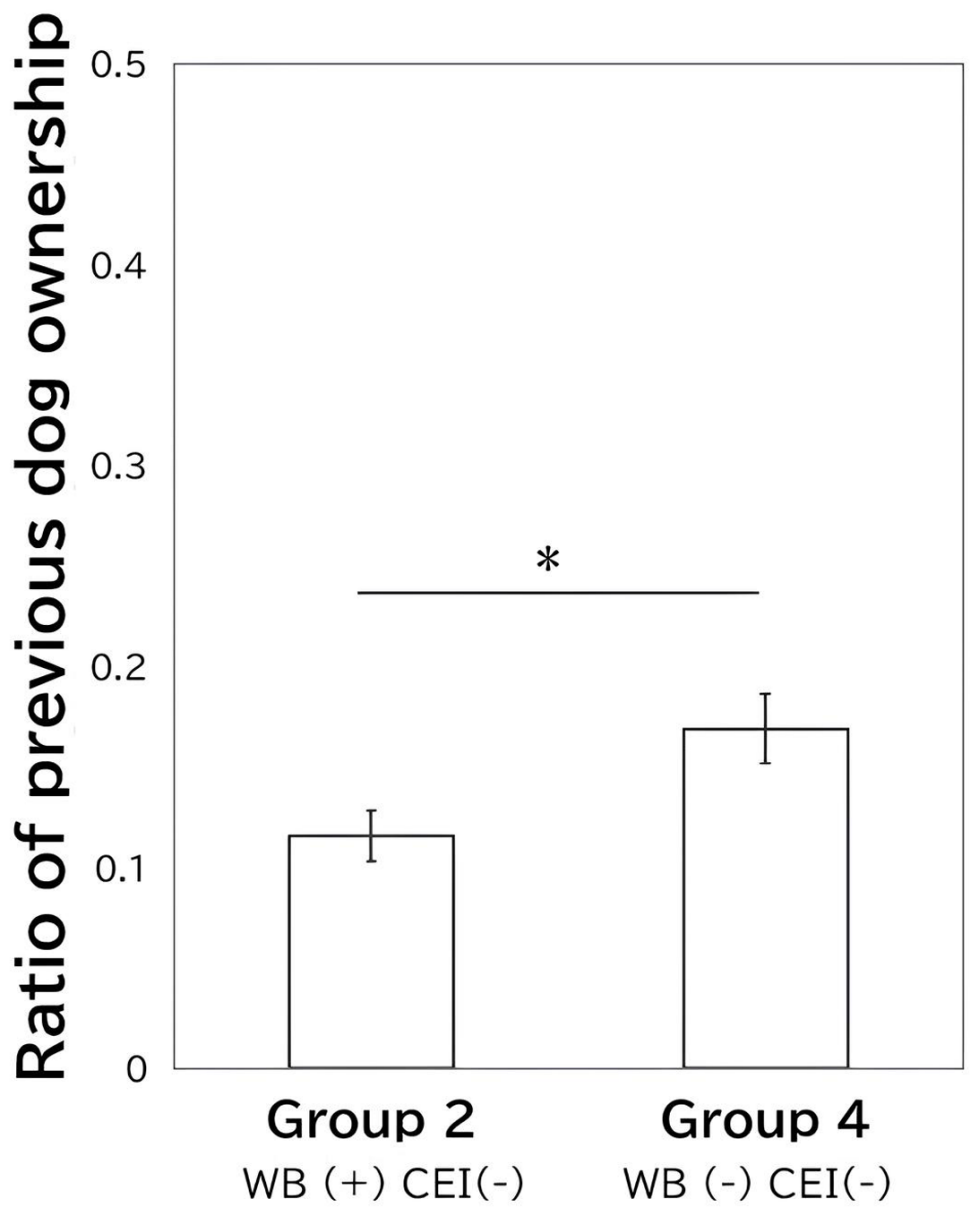
The difference between Groups 2 and 4 in previous dog ownership. Group 2 comprised those with low cultural estrangement and high wellbeing, while Group 3 comprised those with low cultural estrangement and low wellbeing. The bars indicate the ratio of previous dog ownership, and error bars indicate the standard errors. CEI indicates cultural estrangement and WB indicates wellbeing.

Regarding the current ownership rates of dogs or cats, no differences were found between the four CEI and wellbeing groups [dog ownership: *χ^2^* (3) = 6.161, *p* = 0.104; cat ownership: *χ^2^* (3) = 3.769, *p* = 0.286]. Additionally, there was no difference between the four groups in previous cat ownership [*χ^2^* (3) = 1.180, *p* = 0.758], but there was a difference in previous dog ownership [*χ^2^* (3) = 8.694, *p* = 0.034]. Based on a *pair-wise chi-square test* of previous dog ownership, Group 2 was higher than Group 4 [Figure 3, *χ^2^* (1) = 9.813, *p* = 0.002].

### 3.3 Attachment to pets

In this study, we focused on dog and cat owners (both current and previous) and compared attachment levels among different groups based on their interactions with pets (see Table 3). Significant group effects were found for both basic and close attachment [basic attachment: *F*(3, 836) = 11.49, *p* < 0.001; close attachment: *F*(3, 836) = 5.76, *p* < 0.001]. Multiple comparisons revealed that Group 2 exhibited higher levels of basic attachment compared to Groups 1, 3, and 4 (all *p* < 0.001). For close attachment, Group 2 scored higher than Group 3, Group 1, and Group 4 (all *p* < 0.001).

**Table 3:** Comparison among the four groups of CEI and wellbeing for basic attachment and close attachment.

Attachment	ANOVA		Multiple comparisons					
	*F* value	*p*	**1 vs 3**	2 vs. 4	1 vs. 2	1 vs. 4	2 vs. 3	3 vs. 4
Basic attachment	11.490	<0.001		2 > 4 **	2 > 1 **		2 > 3 **	4 > 3 **
Close attachment	28.891	<0.001	1 > 3 **		1 > 2 **	1 > 4 **		3 > 4 **

Multiple comparison results show comparisons between groups (**p < 0.001). Bold text indicates comparisons between Groups 1 and 3, which were the focus of this study in particular.

To further investigate the relationship with pets, we examined responses to each attachment-related item (see Table 4). *ANOVA* revealed significant effects for 10 out of the 12 items. For example, Group 2 scored higher on items such as “My pet makes me feel happy” compared to Groups 3 and 4 (*p* = 0.003 and *p* = 0.001, respectively), and on “I do not want to take care of my pet as much as possible,” Group 2 also had higher scores than Groups 1 and 3 (*p* = 0.002 and *p* < 0.001). On the other hand, Group 2 scored lower on “I often talk to my pet” compared to Groups 3, 4, and 1 (all *p* < 0.001), and “I sleep with my pet” showed significantly lower scores for Group 2 compared to Groups 3, 4, and 1 (all *p* < 0.001).

**Table 4:** Comparison among the four groups of CEI and wellbeing for each attachment item.

Attachment items	ANOVA		Multiple comparisons					
	*F* value	*p*	1 vs 3	2 vs. 4	1 vs. 2	1 vs. 4	2 vs. 3	3 vs. 4
I feel relaxed when I’m with my pet.	7.68	0.053						
My pet makes me feel happy.	13.38	0.004		2 > 4 *			2 > 3 *	
I do not want to take care of my pet as much as possible.	23.69	<0.001			1 < 2 *		2 > 3 **	
Having a pet is a waste of money.	21.57	<0.001			1 < 2 **		2 > 3 **	
I often talk to my pet.	31.13	<0.001		2 < 4 **	1 > 2 **		2 < 3 **	
I sleep with my pet.	29.88	<0.001		2 < 4 **	1 > 2 **		2 < 3 **	
I always talk about important things or open my heart to my pet.	10.03	0.018	1 > 3 **					
I feel closer to my pet than to any of my family members.	16.77	<0.001		2 > 4 **		1 > 4 *	2 > 3 *	
Even when I’m out, I always worry about my pet and hurry home.	19.07	<0.001		2 > 4 **			2 > 3 **	
I like to dress up my pet.	8.38	0.039				1 > 4 *		
I always have a picture of my pet with me.	13.86	0.003		2 > 4 *			2 > 3 **	
Sometimes I feel like my pet is my best friend.	3.33	0.344						

Multiple comparison results show comparisons between groups (*p < 0.05, **p < 0.001). Bold text indicates comparisons between Groups 1 and 3, which were the focus of this study in particular.

**Figure 4:**
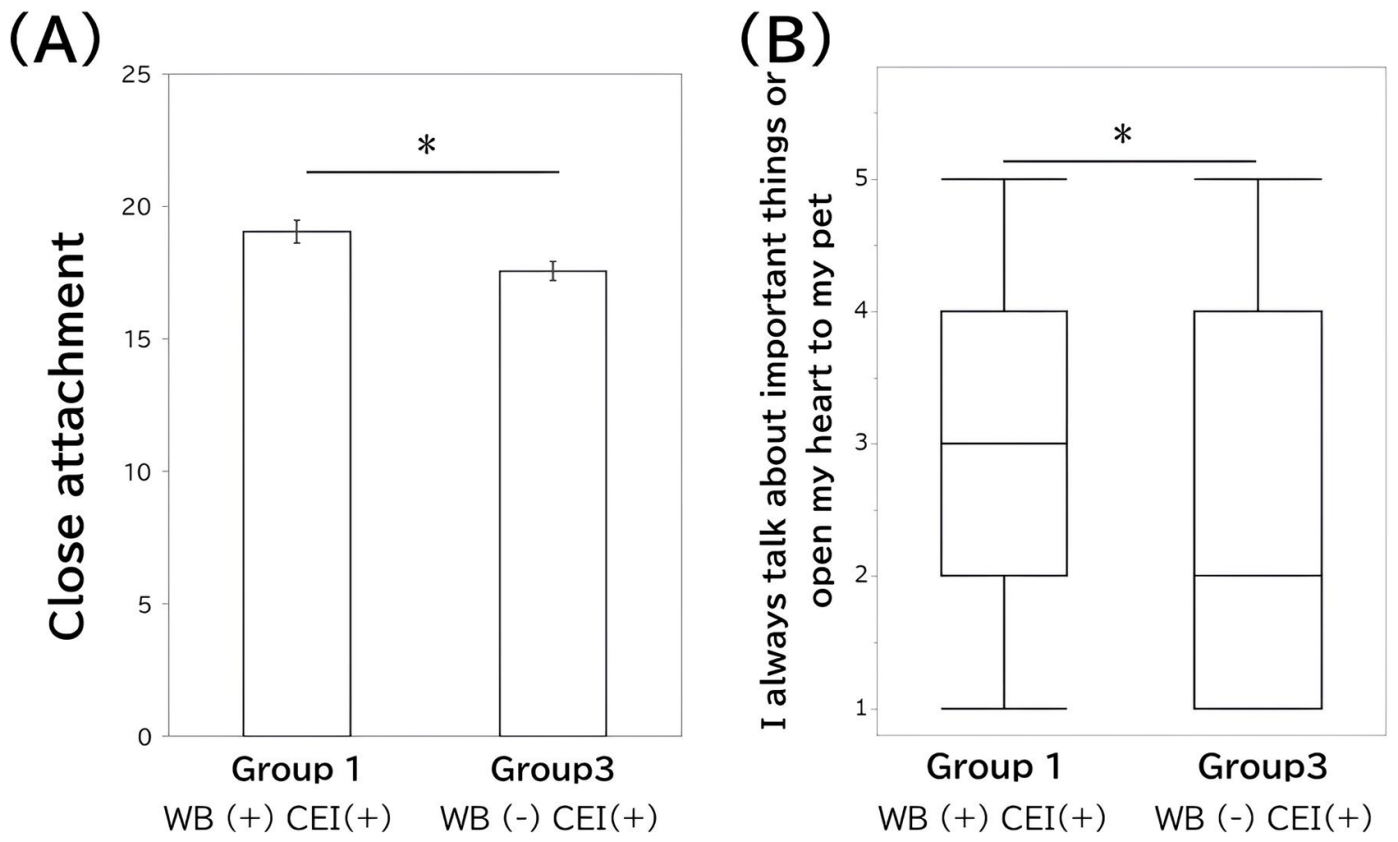
Differences between Groups 1 and 3 in close attachment and “I always talk about important things or open my heart to my pet.” Group 1 comprised those with high cultural estrangement and high well-being, while Group 3 comprised those with high cultural estrangement and low well-being. **(A)** Shows close attachment by the bar plot (error bars indicate the standard errors), while **(B)** shows the attachment item “I always talk about important things or open my heart to my pet” by the box plot. CEI indicates cultural estrangement and WB indicates wellbeing.

Additionally, for the item “I feel closer to my pet than to any of my family members,” Group 2 scored higher than Groups 3 and 4 (*p* < 0.001), and Group 1 also had higher scores than Group 4 (*p* = 0.002). For “Even when I am out, I always worry about my pet and hurry home,” Group 2 scored higher than Groups 3 and 4 (both *p* < 0.001). To summarize, comparing Groups 1 and 3, Group 1 showed higher close attachment overall (see Figure 4A), with a notably higher score for the item “I always talk about important things or open my heart to my pet” (Figure 4B).

## 4 Discussion

This study investigated the differences in the relationship with companion animals among the four groups of cultural estrangement and wellbeing, especially the group with high cultural estrangement and high wellbeing (Group 1) and the group with high cultural estrangement and low wellbeing (Group 3). The results showed that the former group had more anthropocentric attitudes and interest in ecology and the environment than the latter group. In addition, attachment to their pets influenced the owner’s levels of wellbeing among those with high levels of cultural estrangement. Meanwhile, the group with high cultural estrangement and high wellbeing showed higher close attachment than the group with high cultural estrangement and low wellbeing. As for the item “I always talk about important things or open my heart to my pet,” it was also higher in the group with high cultural estrangement and high wellbeing.

Maintaining high wellbeing despite high cultural estrangement is expected to be supported by having self-esteem or having a confidant. Although there are many definitions highlighting different aspects (e.g., Rosenberg, 1965; Sedikides and Gregg, 2007; Murphy et al., 2005; Wang and Ollendick, 2001; Brown et al., 2001), self-esteem can be described as an overall sense of self-worth and personal value. Companion animals are one supportive aspect of self-esteem. For example, it has been shown that children who grew up with pets have higher self-esteem than those who did not (Houtte and Jarvis, 1995). A systematic review of pet ownership and self-esteem suggests that attachment is a significant factor (Purewal et al., 2017). High self-esteem may help them to be self-confident in their own values and not perceive discrepancies with environment negatively.

Confidants provide the opportunity to talk about their concerns (Cornwell et al., 2009; Hall et al., 2020). This not only provides emotional support but also has a significant preventive effect on mental health (Santini et al., 2015; Rueger et al., 2016; Nishida et al., 2024). When ambivalence over emotional expression is high, individuals with close attachment to their pets have been shown to recognize more social support than those with low attachment (Bryan et al., 2014). In other words, companion animals cannot directly solve problems that an individual is facing in society, but they can provide emotional support.

While relationships with companion animals have similarities to interpersonal relationships, a unique aspect also exists in that they provide unconditional acceptance (Hamano, 2007). They may also serve as an easily accessible and non-judgmental audience for emotional disclosure, contributing to an individual’s mental health. In addition, their presence can facilitate conversations in the family (Tannen, 2004; Reider et al., 2023; Koyasu et al., 2023), fostering a close relationship among family members and providing psychological support. The experience of being accepted by others can also create stability in an individual’s self-perception and orient him/her toward internal motivation that is unrestrained by external social values and standards such as peer relations (Deci and Ryan, 1995).

In this study, people with high cultural estrangement and high wellbeing tended to confide in their pets while also exhibiting an interest in nature and an anthropocentric attitude. This suggests they use animals both as “substitutes for social connections” to maintain wellbeing and as “resources” for survival and convenience. One possible reason is that they clarify their attitudes toward the outside world while forming strong attachments to pets but maintaining a utilitarian perspective on other animals. Our findings suggest that individuals may benefit from a multi-faceted strategy in their relationships with animals. To our knowledge, no study has comprehensively examined attitudes toward animals and mental health. A further in depth exploration of attitudes toward animals through qualitative data investigation such as interviews may be the key to solving this question. Another possibility is that interactions in which pets are substitutes for human-human relationships, such as confidants, can be considered anthropocentric in some sense. People treat a pet as a projection or extension of themselves and build their relationship according to their own values. The individual characteristics underlying the two attitudes might be common. Examining the relationship between personality traits and attitudes toward animals could provide insights.

Regarding ownership status, the group with low cultural estrangement and low wellbeing was higher in previous dog ownership than the group with low cultural estrangement and high wellbeing. The loss of a companion animal is significant (Walsh, 2009; Carmack, 2016), since it can affect an individual’s emotional and mental health in a similar manner to the loss of a close family member or friend (Archer and Winchester, 1994; Cleary et al., 2022; Gerwolls and Labott, 1994). Related research has found that pet bereavement in childhood is associated with subsequent psychiatric disorders (Crawford et al., 2021). For example, the loss of a companion animal during adolescence is often the first bereavement experience, which has a significant impact on emotional development and perspectives of life and death. Finally, although the age and duration of pet ownership were not examined in this study, future research should examine the effects of age and the degree of involvement with companion animals in more detail.

Overall, this study includes some limitations that should be noted. First, since it was a cross-sectional study, it did not provide an actual causal relationship. Thus, a longitudinal study of the mental health of adolescents, whose attitudes toward animals and attachment have significantly changed (due to certain life experiences), can provide a clearer understanding of the causal relationship between relationships with companion animals and mental health. In addition, it is necessary to consider the perspective of the animal side and also investigate how animals actually provide support. The second limitation is the possible gender bias in this study. Specifically, although approximately three-fourths of the data was based on the responses of females, there was not a strong gender bias among the four groups. However, future research should improve sampling procedures, such as stratified random sampling, in order to reduce the bias of demographic attributes. Additionally, as for attitudes toward animals, they were categorized into three factors in this study. However, since wide classifications may have masked diversity, a more detailed analysis is necessary to examine the relationship between adolescents’ attitudes toward animals and mental health. These analyses should be done on a new sample different from this research. In this survey, respondents are strictly screened by the research company to check for duplicate answers. However, since this is an online self-response questionnaire, the reliability of the responses cannot be completely ensured. This research targeted only Japanese adolescent. It is expected that future surveys will be conducted on a variety of subjects to identify mental health supports appropriate to each culture and generation.

In conclusion, it was found that those with high cultural estrangement and high wellbeing had more anthropocentric attitudes and interest in ecology and nature, but also more close relationships with their companion animals, especially in self-disclosure such as opening their hearts and talking about important matters. This study was one piece of evidence suggesting that a companion animal supports adolescent’s mental health by encouraging self-disclosure.

## Data Availability

The datasets presented in this article are not readily available because approval for opening all data to the public has not been obtained from the participant. Requests to access the datasets should be directed to the corresponding author.
