# Corrigendum: Study on adolescents' attitudes and attachment toward companion animals: mitigating the negative effects of cultural estrangement on wellbeing

**DOI:** 10.3389/fpsyg.2025.1629552

**Published:** 2025-06-09

**Authors:** Hikari Koyasu, Sakura Ogasawara, Takefumi Kikusui, Toshiya Murai, Atsushi Nishida, Miho Nagasawa

**Affiliations:** ^1^Laboratory of Human-Animal Interaction and Reciprocity, Department of Animal Science and Biotechnology, Azabu University, Sagamihara, Kanagawa, Japan; ^2^Department of Psychiatry, Graduate School of Medicine, Kyoto University, Kyoto, Japan; ^3^The Unit for Mental Health Promotion, Research Center for Social Science & Medicine, Tokyo Metropolitan Institute of Medical Science, Tokyo, Japan

**Keywords:** mental health, cultural estrangement, adolescent, companion animals, attachment

In the published article, there was an error in Table 4 as published. Several statistically significant differences were missing, and one inequality sign was incorrect. The corrected Table 4 based on the original main text and statistical results, and its caption appears below.

**Table 4:** Comparison among the four groups of CEI and wellbeing for each attachment item.

**Attachment items**	**ANOVA**		**Multiple comparisons**					
	***F*** **value**	***p***	**1 vs. 3**	**2 vs. 4**	**1 vs. 2**	**1 vs. 4**	**2 vs. 3**	**3 vs. 4**
I feel relaxed when I'm with my pet	7.68	0.053						
My pet makes me feel happy	13.38	0.004		2 > 4^*^			2 > 3^*^	
I don't want to take care of my pet as much as possible	23.69	< 0.001			1 < 2^*^		2 > 3^**^	
Having a pet is a waste of money	21.57	< 0.001			1 < 2^**^		2 > 3^**^	
I often talk to my pet	31.13	< 0.001		2 < 4^**^	1 > 2^**^		2 < 3^**^	
I sleep with my pet	29.88	< 0.001		2 < 4^**^	1 > 2^**^		2 < 3^**^	
I always talk about important things or open my heart to my pet	10.03	0.018	**1** **>** 3^**^					
I feel closer to my pet than to any of my family members	16.77	< 0.001		2 > 4^**^		1 > 4^*^	2 > 3^*^	
Even when I'm out, I always worry about my pet and hurry home	19.07	< 0.001		2 > 4^**^			2 > 3^**^	
I like to dress up my pet	8.38	0.039				1 > 4^*^		
I always have a picture of my pet with me	13.86	0.003		2 > 4^*^			2 > 3^**^	
Sometimes I feel like my pet is my best friend	3.33	0.344						

Multiple comparison results show comparisons between (^*^p < 0.05, ^**^p < 0.001). Bold text indicates comparisons between Groups 1 and 3, which were the focus of this study in particular.

The authors apologize for this error and state that this does not change the scientific conclusions of the article in any way. The original article has been updated.

